# A Novel ceRNET Relying on the lncRNA JPX, miR-378a-3p, and Its mRNA Targets in Lung Cancer

**DOI:** 10.3390/cancers16081526

**Published:** 2024-04-17

**Authors:** Nicola Mosca, Mariaceleste Pezzullo, Ilenia De Leo, Anna Truda, Giovanna Marchese, Aniello Russo, Nicoletta Potenza

**Affiliations:** 1 Department of Environmental, Biological and Pharmaceutical Sciences and Technologies, University of Campania “Luigi Vanvitelli”, 81100 Caserta, Italy; nicola.mosca@unicampania.it (N.M.); mariaceleste.pezzullo@unicampania.it (M.P.); ilenia.deleo@unicampania.it (I.D.L.); anna.truda@unicampania.it (A.T.); aniello.russo@unicampania.it (A.R.); 2 Genomix4Life S.r.l., 84081 Baronissi, Italy; giovanna.marchese@genomix4life.com; 3 Genome Research Center for Health—CRGS, 84081 Baronissi, Italy

**Keywords:** ncRNA, ceRNET, lncRNA, JPX, miRNA, lung cancer, NSCLC, LUAD

## Abstract

**Simple Summary:**

Non-coding RNAs, particularly microRNAs (miRNAs) and long non-coding RNAs (lncRNAs), are emerging as a driving force for lung cancer, the leading cause of cancer-related death. In this experimental work, we unveiled a novel competing endogenous RNA network (ceRNET) involving the lncRNA JPX, miR-378a-3p, and its downstream oncogenic targets in lung adenocarcinoma cells. First, a reverse expression pattern of JPX and miR-378a-3p was found in tumor tissues compared to normal lungs; subsequently, physical interaction between the molecules was demonstrated. Then, the boosting of either JPX or/and miR-378a-3p levels in lung cancer cells demonstrated the oncogenic role of JPX, the oncosuppressive function of the miRNA, and their functional relationship using an array of biological assays evaluating cell proliferation, migration, invasion, and 3D-spheroid formation. Finally, the ability of JPX to inhibit the silencing activity of miR-378a-3p toward its targets GLUT1, NRP1, YY1, and Wnt5a, and thus the contribution to lung cancer, was demonstrated.

**Abstract:**

Lung cancer is the leading cause of cancer-related death worldwide. Non-coding RNAs are emerging as critical players for the onset and progression of cancer. Analyses of three different datasets revealed that the lncRNA JPX was overexpressed in adenocarcinoma tissues in comparison to normal lungs, as expected for an oncogene. Intriguingly, the predicted binding miR-378a-3p showed a significant inverse correlation with JPX expression. The lncRNA/miRNA physical interaction was validated by reporter vectors. Then, the oncogenic activity of JPX, the tumor-suppressive role of miR-378a-3p, and the contribution of their functional interaction to cancer hallmarks were demonstrated using assays for cell proliferation, migration, invasion, and 3D-spheroid formation. Finally, molecular circuits were investigated by boosting the expression of both JPX and miR-378a-3p, singularly and in combination, demonstrating that JPX counteracted miR-378a-3p silencing activity toward its oncogenic targets GLUT1, NRP1, YY1, and Wnt5a. Overall, the data unveil a novel ceRNET (competing endogenous RNA network), wherein JPX acts as a ceRNA by binding to miR-378a-3p, thus reducing the miRNA silencing activity toward its downstream targets, and eliciting oncogenic pathways driving lung cancer. The knowledge of the network may pave the way to develop new diagnostic panels, and innovative RNA-targeted and RNA-based therapeutic strategies.

## 1. Introduction

Lung cancer (LC) is one of the most common and malignant types of cancer. It is the leading cause of cancer-related mortality with 1.8 million deaths worldwide; although significant advances in early diagnosis and treatment have been made, the prognosis still remains largely unsatisfactory, with a 5-year survival rate of less than 15% [1]. Non-small cell lung cancer (NSCLC) represents the most common histological type with 80–85% of cases in comparison to 15–20% cases of small-cell lung cancer (SCLC); lung adenocarcinoma (LUAD) represents the most common subtype of NSCLC with 50–60% of cases, and remaining cases reported as squamous cell carcinomas (SCCs) [2].

The risk factors are well known, including tobacco smoking, air pollution, viruses, as well as driver mutations; the most frequently mutations found in clinical practice are those in EGFR and the translocation of the ALK gene; in addition, mutations in ROS1, RET, BRAF, HER2, MET, RB1, and TP53 are known to contribute to lung cancer progression [3,4].

Recently, the role of non-coding RNAs in lung cancer appeared on the scene, particularly microRNAs (miRNAs), among the ncRNAs smaller than 200 nucleotides, and long non-coding RNAs (lncRNAs), with a size up to several kilobases [5]. It is becoming increasingly clear that they play a key role in lung cancer, by acting as oncogenes, triggering the molecular pathways leading to cancer onset and progression, but also as tumor suppressors, by braking the cancer hallmarks; in addition, they can even have a role in therapeutic responses such as chemoresistance [6,7,8].

Mechanistically, miRNAs work by driving multiprotein complexes on complementary sequences of target transcripts, thus affecting their translation and/or stability, and indeed regulating gene expression at the post-transcriptional level [9,10]. lncRNAs, due to their intricate and versatile structure, are able to bind to DNA, other RNA biotypes, and proteins, thus modulating gene expression at the epigenetic level, transcription level, post-transcriptional level, and at the translational and post-translational level [11].

In particular, many studies are being published on the ceRNA (competing endogenous RNA) activity of lncRNAs: the lncRNA can bind to a miRNA and, titrating its availability, can endogenously compete with the other miRNA targets, coding or non-coding RNAs, that become de-regulated [12,13,14]. In this view, all RNA biotypes, independently from their coding potentiality, can reciprocally fine-tune their expression levels by competing for binding to a shared pool of miRNAs, thus depicting regulatory networks, indicated as ceRNETs (competing endogenous RNA networks), which are responsible for managing various physiological pathways, and the unbalancing of which can drive carcinogenesis [14,15]. The unifying picture provided by those large-scale networks based on RNA molecules, whose “letters” are the miRNA binding sites of an “RNA code”, has assigned unexpected functions to molecules involved in different biological processes [12]. This is the case of XIST, the essential factor for the inactivation of one X chromosome in mammalian females, and whose study is recently becoming dominated by miRNA association and ceRNA mechanisms, thus involving the lncRNA in different diseases, especially cancer [16]. 

JPX (Just proximal to XIST) is a long non-coding RNA transcribed from the nearest gene located upstream of XIST (~10 kbp), expressed in the antisense direction, acting as a positive regulator of XIST, and thus a key molecular switch for X chromosome inactivation [17]. Sixteen results were retrieved by searching “(JPX) AND (miRNA)” throughout PubMed, very few in comparison to the 428 documents retrieved from the same research on XIST; however, those results indicate its prevailing role as an oncogene in cancer, such as in oral squamous cell carcinomas, gastric cancer, and lung cancer, similarly to those reported for XIST [16,18,19,20,21,22]. 

In this work, we discovered a novel ceRNET driven by JPX and involving miR-378a-3p and its different mRNAs targets, eliciting shared oncogenic pathways, thus consistently and ultimately refining the oncogenic role of JPX in lung cancer, particularly in LUAD, the most common subtype of NSCLC.

## 2. Materials and Methods

### 2.1. DNA Constructs

The JPX overexpressing vector was prepared by synthesizing the JPX sequence NR_024582.1 with additional 5′ *Eco*RI and 3′ *Xho*I restriction sites (Synbio Technologies, Monmouth Junction, NJ, USA) and by cloning it into the same restriction sites of the pcDNA3.1 (+) vector (Thermo Fisher Scientific, Waltham, MA, USA).

Luciferase reporter constructs were prepared as follows: the JPX sequence potentially targeted by the miRNA was obtained using the chemical synthesis of complementary oligonucleotides (Invitrogen, Waltham, MA, USA) including upstream *Xho*I and *Eco*RV restriction sites and a downstream *Not*I site; once annealed, the couple of oligonucleotides were ligated into *Xho*I and *Not*I restriction sites of the psiCheck-2 vector (Promega, Madison, WI, USA) [23]. Recombinant clones, denominated WT-JPX378, were identified by *Eco*RV digestions. The same approach was used to obtain the control plasmid (indicated as I-JPX378), with the exception that the cloned couple of oligonucleotides represented the inverted target sequence. Sequencing was used to confirm the identity of all the constructs.

### 2.2. Cell Cultures, Transfections, and Luciferase Assays

The human lung adenocarcinoma cell line A549 (ATCC, Manassas, VA, USA) was cultured in DMEM supplemented with 10% fetal bovine serum, 50 U/mL penicillin, and 100 μg/mL streptomycin; the human lung adenocarcinoma cell line Calu-3 (ATCC, Manassas, VA, USA) was cultured in MEM with 10% fetal bovine serum, non-essential amino acids (NEAA 1x), 1 mM sodium pyruvate, 50 U/mL penicillin, and 100 μg/mL streptomycin. 

The cells were trypsinized the day before transfection and seeded in a medium without antibiotics in 12-well plates.

Transfections were performed with cells at 80–90% of confluence by using 3 μL of Lipofectamine 2000 (Invitrogen, Thermo Fisher Scientific) for 1 μg of nucleic acids, as described by the manufacturer. In particular, cells were transfected with 200 ng of reporter constructs (WT-JPX378 and I-JPX-378); the miR-378a mimic and its control (Ctrl-miR) with unrelated sequence (Dharmacon, Lafayette, CO, USA) were transfected at 50 nM; 1.2 μg of JPX overexpressing vector (JPX-OV) or the parental plasmid pcDNA3.1 (V) were used for transfection performed on 12-well plates, and 120 ng of both plasmids were used for transfections on 96-well plates. The transfection mix was replaced with complete medium after 6 h, and the analyses were performed 48 h after transfection.

Luciferase assays were performed using the Dual-Luciferase Reporter Assay System (Promega) according to the manufacturer’s protocol.

Experiments were independently repeated at least two times in triplicates.

### 2.3. Cell Proliferation, Cell Migration, and Invasion Assays

Cell proliferation was evaluated using the MTT assay. In brief, cells were plated in 96-well plates, transfected as detailed above, and 48 h later their growth was evaluated by adding 150 μL of medium with 0.5 mg/mL 3-(4,5-Dimethylthiazol-2-yl)-2,5-Diphenyltetrazolium bromide (MTT) to each well; 1 h after incubation at 37 °C, the medium was discarded, and the purple formazan crystals that generated in the viable cells were solubilized in 100 μL of dimethyl sulfoxide and quantified with absorbance measurement at 570 nm with a plate reader.

Cell migration was evaluated using the wound-healing assay. In brief, cells were transfected in 12-well plates as detailed above. When the cells reached the confluence, a sterile 200-μL pipette tip was used to create a uniform scratch on the plate. The cells that detached due to the scratch were subsequently washed away with PBS, and a fresh serum-free culture medium was added. Empty-space colonization by cells was imaged at 0 and 48 h with a microscope and quantifications were performed using Image J 1.52a.

Cell invasion was evaluated using the transwell assay. In brief, cells were transfected in 12-well plates as detailed above and 24 h later were plated in the upper transwell compartment, which was precoated with Geltrex (Gibco, Thermo Fisher Scientific) and filled with a serum-free medium; the lower chamber was filled with medium containing 10% FBS. Sixteen hours later, the invaded cells were fixed with 4% PFA, and then stained using crystal violet. The images of five fields at random were captured via microscope and cell counting was performed using Image J 1.52a.

### 2.4. 3D-Sheroid Formation Assay

A549 cells were transfected on 12-well plates as detailed above, and 24 h after transfection 5 × 10^3^ cells/well were seeded in a BIOFLOAT™ 96-well cell culture plate (faCellitate, Mannheim, Germany) according to the manufacturer’s recommendations. The ability of cells to form spheroids was evaluated by daily monitoring the cultures under the microscope and acquiring images of the floating spheres. On day 5, the spheroids were dissociated by trypsinization, and the cells were counted.

### 2.5. RNA Purification and Real-Time PCR Analyses

Total RNA was extracted from cell cultures using the miRNeasy mini kit (Qiagen, Hilden, Germany). MicroRNA-378a was quantified along with RNU6B (reference transcript) by RT-qPCR with TaqMan^®^ miRNA assays (Applied Biosystems, Waltham, MA, USA) according to the manufacturer’s protocol.

For quantification of the other transcripts, total RNA was retrotranscribed using the SensiFAST cDNA Synthesis kit (Bioline, London, UK). Then standard SYBR Green Real-time qPCR assays were performed with the following primers:

JPX, 5′-TGCAGTCAGAAGGGAGCAAT-3′ and 5′-CACCGTCATCAGGCTGTCTT-3′ [24];

IGF1R, 5′-CAAGCCTGAGCAAGATGATTC-3′ and 5′-GAACTTATTGGCGTTGAGGTATG-3′ [25];

GLUT1, 5′-AAGGTGATCGAGGAGTTCTACA-3′ and 5′-ATGCCCCCAACAGAAAAGATG-3′ [26];

Wnt5a, 5′-CAAGGGCTCCTACGAGAGTG-3′ and 5′-CCCACCTTGCGGAAGTCT-3′ [27];

YY1, 5′-ACGGCTTCGAGGATCAGATTC-3′ and 5′-TGACCAGCGTTTGTTCAATGT-3′ [28];

DCTPP1, 5′-CGCCTCCATGCTGAGTTTG-3′ and 5′-CCAGGTTCCCCATCGGTTTTC-3′ [29];

GOLT1A, 5′-GGGCCTGTCCCTCATCATT-3′ and 5′-TTTGTGCCGTTGGAAGAAGAA-3′ [30];

cRaf, 5′-GCAATGAAGAGGCTGGTAGC-3′ and 5′-GGAGCAGCTCAATGGAAGAC-3′ [31];

VEGFR, 5′-TTTGCCTGAAATGGTGAGTAAGG-3′ and 5′-TGGTTTGCTTGAGCTGTGTTC-3′ [32];

NRP1, 5′-GGCGCTTTTCGCAACGATAAA-3′ and 5′-TCGCATTTTTCACTTGGGTGAT-3′ [33];

GAPDH (reference transcript), 5′-GAAGGTGAAGGTCGGAGTC-3′ and 5′-GAAGATGGTGATGGGATTT-3′.

The expression levels of miRNAs and transcripts were normalized to their respective reference genes by using the 2-DCt method.

### 2.6. Statistical and Bioinformatic Analyses

All data are reported as the mean of three or more independent experiments and error bars indicate standard deviation (SD) of the mean. Statistical analyses were performed using GraphPad Prism 9.1 software. Comparison of datasets in the different experiments was performed using the Student’s *t*-test and a value of *p* < 0.05 was considered statistically significant.

Transcriptomic data were analyzed from R2 (Genomics analysis and visualization platform, https://r2.amc.nl accessed on 10 July 2023) or the ENCORI platform (The Encyclopedia of RNA Interactomes, https://rnasysu.com/encori accessed on 10 July 2023). In particular, the microarray datasets GSE19188 and GSE33532 were downloaded from R2 and filtered to select lung adenocarcinoma samples and normal lung tissues. The ENCORI platform was exploited to analyze expression data from patients based on the expression values of genes and miRNAs from RNA-seq and miRNA-seq data of The Cancer Genome Atlas (TCGA) [34]. In brief, in the section Pan-Cancer of the ENCORI platform, we obtained the fold change expression and *p* values between tumor tissues compared to normal lungs from the two subsections “miRNA differential expression” and “Gene differential expression”, for miR-378a and for JPX, respectively. Pearson correlation coefficients were obtained from “miRNA-Target Co-Expression”, where expression values of JPX were scaled with log2 (FPKM + 0.01) and miR-378a scaled with log2 (RPM + 0.01).

## 3. Results

### 3.1. JPX Is Upregulated in Lung Tumor Tissues

**Figure 1:**
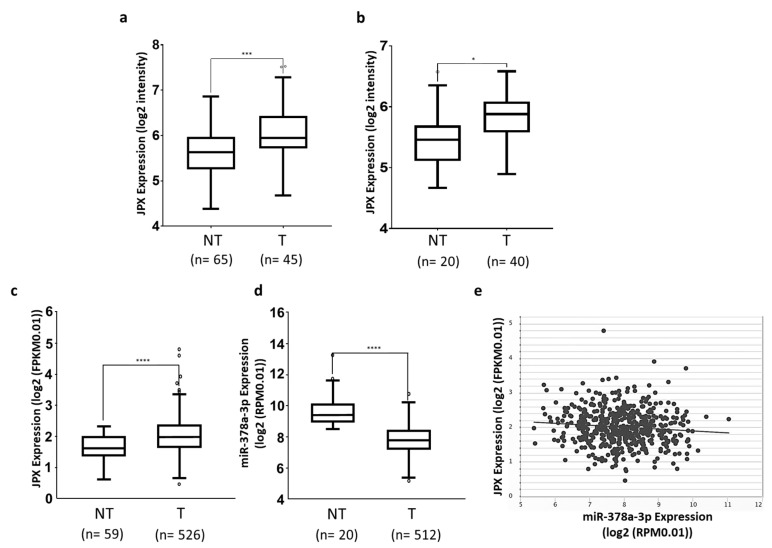
Expression of JPX and miR-378a-3p in lung adenocarcinoma. Expression of JPX in lung adenocarcinoma (T) and non-tumoral samples (NT) from (**a**) GSE19188 and (**b**) GSE33532 datasets from microarray data in R2 platform (https://r2.amc.nl accessed on 10 July 2023). (**c**) LUAD dataset from RNAseq data in TCGA from ENCORI platform (https://rnasysu.com/encori accessed on 10 July 2023). (**d**) miR-378a-3p expression in lung adenocarcinoma and non-tumoral samples in the LUAD dataset from TCGA. (**e**) Graphic representation of inverse correlation between JPX and miR-378a expression in the LUAD dataset from TCGA (r = −0.1, *p* = 2.61 × 10^−2^). Number (n=) of samples is indicated. *p*-values at Student’s *t*-test were * *p* < 0.05, *** *p* < 0.001, or **** *p* < 0.0001.

Different datasets have been exploited to assess the expression level of JPX in tumor tissues in comparison to normal lungs. We downloaded and analyzed the sequencing data of GSE19188 and GSE33532 datasets from R2 and found that JPX was significantly upregulated in lung adenocarcinoma tissues in comparison to normal lungs, with a fold change of 1.45 (*p* = 0.0001) and 1.21 (*p* = 0.0103), respectively (Figure 1a,b). Then, we analyzed data from the very large patient cohort of The Cancer Genome Atlas (TCGA) database and found similar results, with an upregulation of 1.38 (*p* = 2.0 × 10^−7^) in tumor tissues in comparison to normal ones (Figure 1c). Taken together, the data indicate that JPX expression is markedly increased in tumor tissues, which is consistent with data that have already reported on smaller patient cohorts, suggesting an oncogenic role of JPX in lung cancer [20,21,22].

### 3.2. miR-378a Is Inversely Correlated to JPX Expression in Lung Tumor and Interacts with JPX

Some miRNAs have been shown to bind to JPX and many others can be predicted to potentially interact with the lncRNA [16]. Among them, we focused our attention on miR-378a-3p (miR-378a), which was predicted by both RNAhybrid 2.2 [35] and miRDB. In fact, it showed a reverse expression pattern in comparison to JPX, being strongly and significantly down-regulated in tumor tissues in comparison to normal lungs, based on the analyses of data from the larger patient cohort in TCGA (fold change = 0.18, *p* = 5.2 × 10^−19^; Figure 1d). Moreover, the Pearson correlation coefficient showed a significant inverse correlation between JPX and miR-378a expression (r = −0.1, *p* = 2.61 × 10^−2^, Figure 1e). These data prompted us to hypothesize a functional interaction between the lncRNA and the miRNA and a role in lung tumorigenesis.

**Figure 2:**
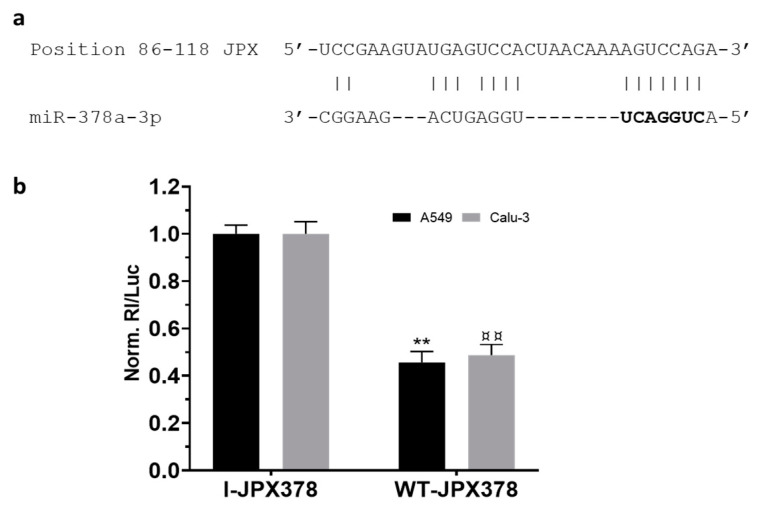
Validation of interaction between JPX and miR-378a. (**a**) Predicted miR-378a binding site on JPX sequence; miRNA seed sequence is marked in bold. (**b**) Cells were transfected with the luciferase-based reporter constructs containing the wild-type target sequence of miRNA (WT-JPX378) or the control plasmids with inverted target sequence (I-JPX378), along with 50 nM miR-378a mimic. Luciferase activities were measured after 48 h; the Renilla luciferase activity (Rl) was normalized to that of firefly luciferase (Luc); the values are reported as fold mean + SD relative to Rl/Luc recorded for control transfection of I-JPX378 plasmid with mimic, which was set to 1. Data are the mean +SD of two independent experiments, each with three datasets. ** or ^¤¤^ *p* < 0.01 at Student’s *t*-test referred to A549 or Calu-3 cells, respectively.

As a first step, the potential binding of miR-378a to JPX was experimentally validated using luciferase reporter vectors transfected in the lung adenocarcinoma cells A549 and Calu-3 (Figure 2). In brief, the JPX-predicted binding site for miR-378a was cloned downstream of the coding sequence of Renilla luciferase as such (WT-JPX378) or inverted as a control (I-JPX378); the vectors were singularly transfected into the cells along with the miR-378a mimic; then, Renilla luciferase activities were recorded as well as the firefly luciferase, the coding sequence of which was carried by the vectors and used as a normalizer. The analyses of data showed more than 50% luciferase inhibition exerted by the miRNA on WT-JPX378 in comparison to the control in both cell lines, thus demonstrating the physical interaction between the miRNA and JPX sequences.

### 3.3. JPX Promotes Cell Proliferation, Migration, and Invasion by Sponging miR-378a

Some studies demonstrated that JPX overexpression was able to prompt cancer hallmarks in cell culture [36,37]. We evaluated this ability in our experimental system, as well as the effects of miR-378a boosting, and those of their combination, by performing an array of biological assays, such as the MTT assay for measuring cell proliferation, wound healing assays for cell migration, and the transwell invasion assay for cell invasion.

**Figure 3:**
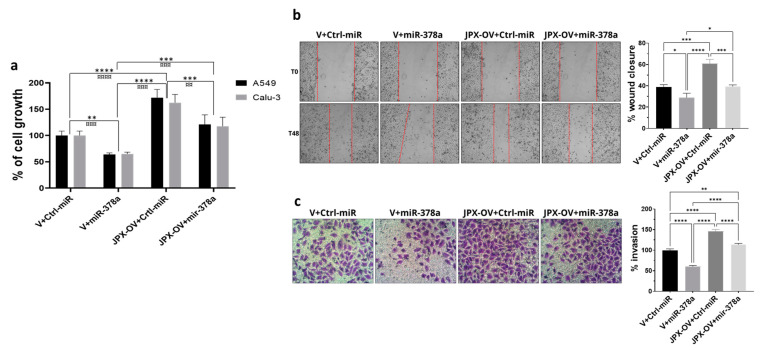
Effect of miR-378a, JPX, and their combination on cell proliferation, migration, and invasion. Cells were transfected with the indicated molecules and 48 h after transfection the different assays were performed. (**a**) Cell growth was evaluated using the MTT assay; the values are reported as a percentage from the transfection control experiment (V+Ctrl-miR), which was set to 100. (**b**) Cell migration was evaluated using the wound-healing assay. Left panel: representative images of migrating cells with the red dotted lines indicating the edge of the gap; Right panel: bar graph of percentage of wound closure relative to transfection control experiment. (**c**) Cell invasion was evaluated using the transwell assay. Left panel: representative images of invading cells, stained by crystal violet; Right panel: bar graph of cell counting percentage of invading cells relative to control transfection experiment (V+Ctrl-miR). Data are the mean ± SD of three independent experiments. * *p* < 0.05, ** or ^¤¤^ *p* < 0.01, *** or ^¤¤¤^ *p* < 0.001, **** or ^¤¤¤¤^ *p* < 0.0001 at Student’s *t*-test, with * and ¤ referring to A549 or Calu-3 cells, respectively. V, parental vector pcDNA3.1; JPX-OV, JPX overexpressing vector; Ctrl-miR, control miRNA mimic; miR-378a, miR-378a-3p mimic.

The transfection of a JPX overexpressing vector (JPX-OV) promoted cell proliferation by 1.7-fold in comparison to the control represented by cells transfected with the parental vector (V); conversely, the boosting of miR-378a obtained by the mimic transfection strongly reduced cell proliferation by 40%. Then, the co-transfection of the JPX-OV and miR-378a mimic demonstrated that JPX overexpression was able to completely revert the inhibitory effect of miR-378a on cell proliferation, bringing it to the level of the transfection control (Figure 3a). From a different point of view, it could be assessed that the effect of JPX overexpression on cell proliferation was significantly mitigated by the increased level of miR-378a.

The same transfection combinations as above were used to evaluate the effect of the two molecules on cell migration and invasion. The transfection of the JPX-OV increased cell migration and invasion by 1.5-fold and 1.4-fold in comparison to the control, respectively; the miR-378 mimic transfection drastically reduced both migration and invasion by 25% and 40%, respectively. Similar to the effect on cell proliferation, the co-transfection of both molecules showed the ability of JPX to abrogate the effects of miR-378a, giving results comparable to transfection controls (Figure 3b,c). From a different point of view, the effect of JPX overexpression was attenuated by the miRNA.

Overall, the results indicate that JPX can exert its oncogenic role by regulating the oncosuppressive effect of the miR-378a, thus promoting cell proliferation, migration, and invasion, and releasing the antitumoral brake represented by the miRNA.

### 3.4. Functional Impact of JPX, miR-378a, and Their Combination on 3D-Spheroid Formation

**Figure 4:**
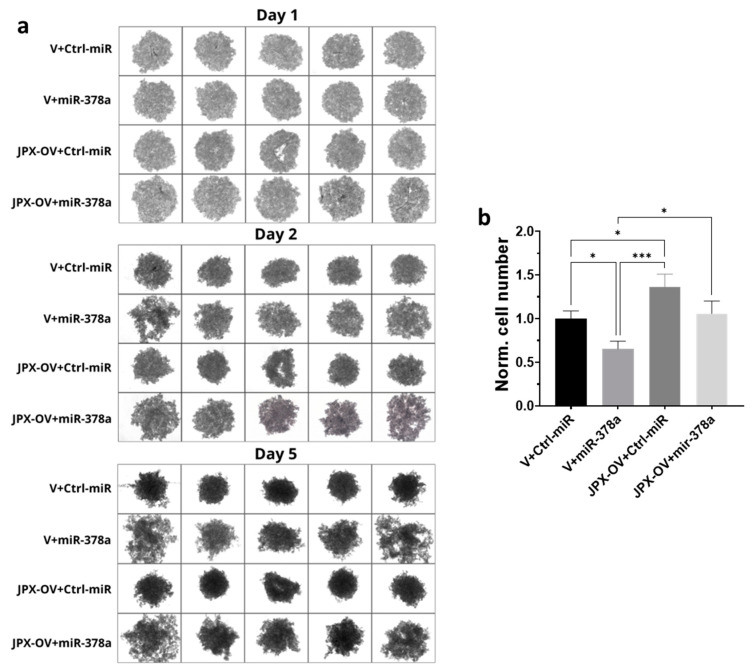
Effect of miR-378a, JPX, and their combination on 3D spheroid formation. A549 cells were transfected with control molecules (V+Ctrl-miR), miR-378a mimic, and JPX-OV, singularly or in combination. The day after transfection, the cells were transferred to 96-well plates and spheroid formation was monitored daily for 5 days. (**a**) Representative images of spheroid formation at day 1, 2, and 5. (**b**) Bar graph of cell counting after trypsinization at day 5; the values are reported as fold mean relative to control transfection experiment (V+Ctrl-miR), which was set to 1. Data are shown as the mean ± SD of three independent experiments. * *p* < 0.05 and *** *p* < 0.001at Student’s *t*-test.

Different cancer cell lines are able to form anchorage-independent aggregations, called spheroids, indicative of their ability to proliferate as cancer stem-like cells and to successfully establish clones with tumor-initiating potential at distant niches for metastasizing [38,39]. The ability to form 3D-spheroids was tested with A549 cells transfected with the control molecules, miR-378a mimic, JPX overexpressing vector, or a combination of both. The control cells formed spheroids that appeared rounder and darker under a microscope, suggestive of an increasing density and cohesivity during the incubation days. The overexpression of miR-378a deeply impacted the spheroid formation, since they appeared as loose and quite unstable aggregates, more severely from day 2 to 5 of incubation. Cells overexpressing JPX showed the ability to form spheroids significantly better than the control, especially after 5 days of culturing. More importantly, its overexpression was able to counteract the loosening effect of the miR-378a mimic (Figure 4a). Although the spheroids had irregular morphologies from the different experimental points, in order to have quantitative data, they were dissociated after culturing for 5 days using trypsinization, and the cells were counted, giving results consistent with the observed phenotypes, i.e., a lower number of cells that make up the spheroids was observed if miR-378a was overexpressed in comparison to control cells; an increased number of cells in spheroids formed by cells overexpressing JPX was observed; and importantly, a similar cell number to the control was observed if JPX was overexpressed in combination with the boosting of miR-378a, thus counteracting the action of the miRNA (Figure 4b).

### 3.5. JPX Counteracts miR-378a Silencing Activity toward Its Oncogenic Targets

Based on the phenotypic features observed in the different assays described above, we hypothesize that JPX can promote cancer hallmarks by reducing the silencing activity of the miRNA toward its targets, competing with them for miRNA binding through the so-called ceRNA mechanism. We explored the literature to find miR-378a experimentally validated targets, related to cancer hallmarks, and selected those consistently reported, although in other type of cancers:

IGF1R, the insulin-like growth factor (IGF) receptor, belongs to the receptor-type tyrosine kinase family, the signaling axis of which is critical for the onset and progression of many tumors; it has been validated as the miR-378a target in glioma, hepatocarcinoma, and rhabdomyosarcoma [40,41,42];

GLUT1, the most widely expressed glucose transporter, is known to act as the basal switch in tumor cell glycolysis and thus in the Warburg effect; it has been validated as the miR-378a target in oral squamous cell carcinoma [43,44];

Wnt5a belongs to the Wnt signaling pathway and has been validated as the miR-378a target in ovarian cancer and papillary thyroid cancer [27,44];

YY1, the transcription factor Yin Yang-1, is an oncogene for various tumors; it has been validated as the miR-378a target in breast and uterine carcinoma [28,45];

DCTTP1, human all-alpha dCTP pyrophosphatase, is upregulated in various cancers and involved in DNA repair signaling, and GOLT1A, Golgi transport 1A, is involved in the ER-to-Golgi network; they have been both validated in breast cancer as the miR-378a target [29,30];

cRAF kinase and VEGFR, vascular endothelial growth factor tyrosine kinase, are key regulators of the MAPK signaling pathway, with an important role in various types of cancer; they have been both validated in liver cancer as the miR-378a target [46];

NRP1, neuropilin 1, is a membrane-bound coreceptor to the tyrosine kinase receptor VEGF, and thus implicated in the vascularization and progression of different cancers; it has been validated as the miRNA target in stomach adenocarcinoma [33].

**Figure 5:**
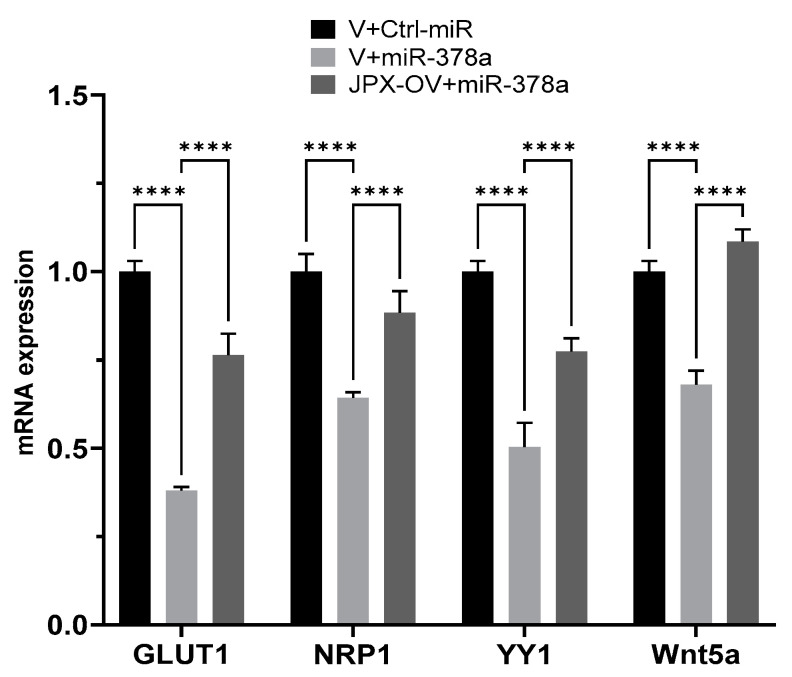
Effect of JPX overexpression on miR-378a silencing activity toward its oncogenic targets. The expression level of the indicated miR-378a targets was evaluated using RT-QPCR on RNA purified from A549 transfected with the indicated molecules. The values are reported as fold mean (2^−∆∆Ct^) relative to control (V+Ctrl-miR). Data are the mean ± SD of replicate experiments. **** *p* < 0.0001 at Student’s *t*-test.

We experimentally verified the inhibitory effect of miR-378a on those targets in lung cancer cells, and the possible ability of JPX in counteracting that effect by competing with the binding to the miRNA. In our experimental system, some targets were not inhibited by the miRNAs (IGF1R, DCTTPP1 and GOLT1A, cRaf and VEGFR) and were excluded for further analyses. Instead, GLUT1, NRP1, YY1, and Wnt5a were strongly inhibited by the miRNA, since the miR-378a transfection reduced them by 30 to 60%; significantly, in the presence of JPX overexpression, the miRNA inhibitory effect was strongly reduced (GLUT1, YY1 and NRP1) or completely reverted (Wnt5a) (Figure 5). These results suggest that JPX works as a ceRNA by effectively binding the miRNA and thus reducing its availability for the silencing of the other targets. Intriguingly, most of those mRNA targets are also upregulated in tumor tissues, particularly GLUT1, which is strongly and consistently overexpressed in LUAD from all datasets (Table 1).

Overall, the results depict a novel ceRNET that can govern different oncogenic pathways, as discussed below.

## 4. Discussion

Lung cancer is the leading cause of cancer-related death worldwide, with NSCLC accounting for the majority of cases (up to 85%), and LUAD representing the most common NSCLC subtype. The contribution of ncRNAs to the onset and progression of LC is becoming increasingly clear, especially for microRNAs and lncRNAs [5]. In addition, the ceRNET perspective has opened a new scenario, wherein even functionally unrelated transcripts can unexpectedly cross talk with each other by competitively binding to a shared microRNA, and new functions have been assigned to molecules involved in distant and unrelated biological processes [13,14]. This is the case of the lncRNA JPX; in recent years, it has been discovered that JPX, beyond serving as an activator of XIST expression essential for X chromosome inactivation, is aberrantly expressed and associated with clinicopathological traits in numerous diseases, particularly cancers, where it acts as an oncogene in the majority of cases (lung cancer, oral squamous cell carcinoma, cervical cancer, osteosarcoma, esophageal squamous cell carcinoma, gastric cancer, ovarian cancer, glioblastoma multiforme, and acute megakaryoblastic leukemia), but also as a tumor suppressor in some others (hepatocellular carcinoma, breast cancer, and uveal melanoma) [36,37]. In lung cancer, JPX was reported to be upregulated in three experiments, where its expression was correlated with clinical characteristics, such as tumor size, stage, and poor survival [16,17,20,21,22]. Our analyses on two small patient cohorts and the larger one of the TCGA gave consistent results, showing an overexpression of JPX in tumor tissues in comparison to normal lungs (Figure 1a–c), as expected for an oncogene.

Mechanistically, some papers reported the ability of JPX to regulate different biological processes involved in oncogenic pathways by ceRNA mechanisms [16,18,19,20,21,22]. Looking for a similar molecular mechanism potentially contributing to LUAD, we noticed that among the predicted microRNAs binding to JPX, miR-378a-3p showed a reverse expression pattern in comparison to JPX, i.e., a strong down-regulation in tumor tissues on the larger patient cohort from the TCGA, and a significant inverse correlation with JPX (Figure 1d,e). Based on those data, we hypothesized a physical interaction between the miRNA and the lncRNA that was indeed experimentally validated (Figure 2).

Thus, in LUAD cell models, an array of biological assays were performed to evaluate the potential oncogenic activity of JPX, the potential tumor-suppressive role of the miRNA, and the contribution of the putative functional interaction of JPX/miR-378a to cancer hallmarks. The cell proliferation assay, wound healing assay, and invasion assay gave consistent results. In particular, JPX promoted cell proliferation, migration, and invasion, confirming data already reported; conversely, miR-378a gave the opposite effect, indicating an oncosuppressive activity; this activity was completely counteracted by JPX overexpression, showing results similar to control experiments (Figure 3). We also decided to explore the functional relevance of JPX/miR-378a interaction by using spheroid models, which are commonly used in cancer research for their ability to better recapitulate the biological and molecular features of the complex tumor architecture, and which thus limit the use of animals for in vivo studies [38,39]. Results that were consistent with the above-described assays were obtained using the 3D-spheroid formation assay. In particular, miR-378a negatively impacted the ability of tumor cells to form anchorage-independent aggregations; however, that ability was recovered when JPX was also overexpressed, again pointing towards the functional relationship between the lncRNA and the miRNA (Figure 4).

**Figure 6:**
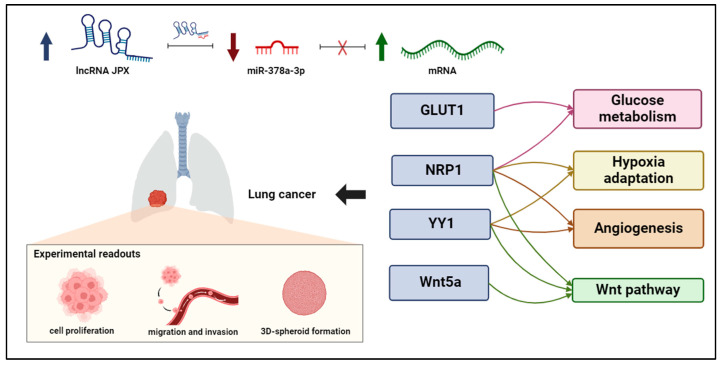
ceRNET relying on JPX, miR-378a-3p, and mRNA targets in lung cancer. Interactions among JPX, miR-378a, and downstream target mRNAs depict a competing endogenous RNA network (ceRNET), since the upregulation of JPX (indicated by arrow up) results in a decreasing availability of miR-378a (indicated by arrow down) for the other mRNA targets, which become upregulated (indicated by arrow up). Intriguingly, the targets validated here are involved in shared oncogenic pathways, indicated on the right. The experimental readouts exploited in this work are indicated. Figure created with BioRender.com.

In order to investigate the molecular circuits underlying the observed phenotypes, we also explored the literature and found that miR-378a is largely reported to be implicated in cancer, with 141 articles retrieved by searching throughout PubMed with key words “378a AND cancer”. Referring specifically to miR-378a-3p in lung cancer, it was consistently reported as a tumor-suppressive miRNA, which is consistent with our experimental data. In particular, it has been reported that the DEAD-box RNA helicase DDX56 promoted squamous cell lung cancer in vitro and in vivo by repressing miR-378a-3p mechanistically through facilitating the degradation of the primary miRNA; a consequent derepression of the Wnt pathway was also reported [47]. Furthermore, miR-378a has been demonstrated to contribute to the mechanism of action of the lncRNA OIP5-AS1, whose oncogenic effect has been demonstrated in vitro and in vivo; in fact, it was verified that OIP5-AS1 was able to sponge miR-378a-3p, thus reverting miRNA antiproliferative activity in lung cancer cells [48]. However, validated downstream miR-378a targets have not been reported in LC; so, we deeply inspected the literature, selecting targets that have been experimentally validated and consistently reported as related to cancer hallmarks, although in other tumor types. As indicated in the Results section, we evaluated those targets in our experimental system and found that GLUT1, NRP1, YY1, and Wnt5a were effectively inhibited by the miRNA in lung cancer cells (Figure 5). Importantly, JPX was able to counteract the silencing activity of the miRNA toward those targets, thus depicting molecular circuits underlying the phenotypic effects observed on cell cultures. In particular, the data indicate that JPX works as a ceRNA, i.e., it competes with the other miRNA targets, unbalancing the regulatory network. Intriguingly, the targets are involved in shared pathways relevant for lung carcinogenesis (Figure 6). In particular, GLUT1 and NRP1 are both involved in glucose metabolism: GLUT1, when overexpressed, accelerates the glucose uptake, thus providing sufficient raw materials for glycolysis; NRP1, if overexpressed, can increase the expression and activity of glycolytic metabolizing enzymes and accelerate glycolysis, thus promoting tumor cell proliferation [43,44,49,50]. In addition, both NRP1 and YY1 are involved in the adaptation of tumor cells to hypoxia, a condition commonly observed in tumor microenvironment due to rapid consumption of oxygen for rapid proliferation and high metabolism [50,51]. Again, they are both involved in promoting angiogenesis, NRP1 as a co-receptor of VEGFR2 and both by stabilizing HIF-1alfa to activate VEGF expression [50,51,52]. Finally, the Wnt pathway, a key player in oncogenesis, is triggered not only by Wnt5a, but also by other targets of the network, i.e., NRP1, and YY1 [27,53,54,55,56].

## 5. Conclusions

The reported data highlight a novel ceRNET, wherein the lncRNA JPX acts as a ceRNA by binding to miR-378a. This interaction reduces the miRNA silencing activity toward its downstream targets, i.e., the mRNAs coding for GLUT1, NRP1, YY1, and Wnt5a, thus contributing to oncogenic pathways driving lung cancer (Figure 6). The knowledge of this RNA regulatory network relying on JPX/miR-378a/mRNAs may pave the way for the development of new diagnostic panels based on the quantification of the involved molecules, but also innovative RNA-targeted and RNA-based therapeutic strategies, by manipulating the network through enhancing or inhibiting the involved molecules.

## Figures and Tables

**Table 1:** Gene expression changes in LUAD tissues compared to normal lung samples from 3 different datasets.

Gene ID	TCGA Log2 Fold Change (*p*-Value)	GSE19188 Log2 Fold Change (*p*-Value)	GSE33532 Log2 Fold Change (*p*-Value)
JPX	1.38 (2.0 × 10^−7^)	1.45 (0.0001)	1.21 (0.0103)
miR-378a-3p	0.18 (5.2 × 10^−19^)	-	-
GLUT1	12.33 (1.8 × 10^−45^)	1.36 (5.61 × 10^−21^)	1.39 (3.44 × 10^−11^)
NRP1	1.02 (ns)	0.93 (9.81 × 10^−6^)	0.96 (2.59 × 10^−3^)
YY1	1.14 (0.0014)	1.04 (6.76 × 10^−11^)	1.06 (9.10 × 10^−13^)
Wnt5a	1.19 (ns)	1.00 (ns)	1.06 (0.012)

-, not reported; ns, not significant.

## Data Availability

The original contributions presented in the study are included in the article; further inquiries can be directed to the corresponding author.
